# *Spartina alterniflora* invasion affects soil carbon in a C_3_ plant-dominated tidal marsh

**DOI:** 10.1038/s41598-017-19111-1

**Published:** 2018-01-12

**Authors:** Min Wang, Qing Wang, Chenyan Sha, Jiakuan Chen

**Affiliations:** 1 Education Key Laboratory for Biodiversity Science and Ecological Engineering, Fudan University, 2005 Songhu Road, Shanghai, 200438 China; 2 Research Institute of Natural Ecology Conservation, Shanghai Academy of Environmental Sciences, 508 Qinzhou Road, Shanghai, 200233 China

## Abstract

The carbon cycle is significantly affected by *Spartina alterniflora* invasion through its impact on blue carbon in many salt marshes. To determine the impacts on soil organic carbon (SOC), we studied the vertical and horizontal distribution of SOC. And stable carbon isotopes were used to explore the impact of the age of *S. alterniflora* invasion on SOC in Chongming Dongtan wetland located in the Yangtze River estuary, China. The results showed that the SOC concentration was higher in the *S. alterniflora* community than that in the native *Phragmites australis* community. The age of invasion and the SOC concentration increased with increasing elevation, while the SOC concentration decreased with increasing soil depth. The δ^13^C value became less negative at greater depth, which was related to the contribution from ^13^C- enriched carbon sources after 3 years of invasion. After 7 and 10 years, the δ^13^C value became more negative at greater depth in both communities. *S. alterniflora* had a positive effect on the soil carbon pool, and its contribution was related to soil depth. In the low tidal marshes, the contribution of *S. alterniflora* was negatively correlated with soil depth, while it was positively correlated with soil depth in the high tidal marshes. The results from this study will contribute to improved understanding of future ecological consequences.

## Introduction

‘Blue carbon’, which refers to carbon sequestered in salt marsh, seagrass, and mangrove ecosystems, is important for understanding the link between terrestrial and oceanic carbon cycles^1–3^. Accounting for all sources of carbon and organic matter contributing to coastal ecosystems is imperative for accurate budgeting of the current global carbon cycle and for forecasting changes in carbon cycling induced by global climate change and other anthropogenic impacts^4,5^, such as land use change. Population changes in native species within the plant community alter the ecosystem structure and the soil carbon pool^6^. Therefore, alterations in the composition of salt marsh plant species, which are affected by current natural and human activities, and their associated carbon sequestration capacity require further attention^4,7,8^.

Plant invasion profoundly influences ecosystem functions and soil processes in ecosystems around the world^6,9,10^. Changes in the dominant species within a plant community may alter ecosystem structures and material cycling processes, which in turn alter above- and belowground carbon pools, net primary productivity, plant growth rates, litter quality and quantity, and nutrient and carbon mineralization rates^11^. Plant invasions mainly influenced soil organic carbon pool by altering the quantity and quality of litter and roots entering the soil. The effect of invasive plants on soil carbon pools is critically important for understanding changes in carbon cycles. However, compared with soil carbon reservoirs, the changes in soil organic matter, even when significant, are small. Therefore, conventional methods cannot be used to examine the variation in the soil organic carbon (SOC) pool within the short period over which most experiments are conducted.

*Spartina alterniflora* was introduced to China from North America in 1979^12^. Since then, this invasive species has rapidly expanded in salt marshes along the coast. However, *S. alterniflora* has negative impacts on native ecosystems, as it continuously encroaches on the habitat of native plant communities, forming a single-dominant plant community^13,14^. Several studies have focused on the effects of *S. alterniflora* invasion on wetland total SOC and the horizontal and vertical distribution and seasonal dynamics of SOC^15^. However, few studies have focused on the effects of *S. alterniflora* on SOC dynamics using an invasion chronosequence.

Stable carbon isotopes have been employed as tracers of the sources of accumulated organic carbon in salt marsh sediments^16–24^. This technique is based on differences in stable carbon isotope ratios, such as those between the soil carbon inputs of *S. alterniflora* (C_4_ source) and *Phragmites australis* (C_3_ source). C_4_ and C_3_ sources are readily distinguished by their distinctive δ^13^C values. C_3_ plants have been reported to exhibit δ^13^C values between −35 and −20‰, while those of C_4_ plants range between −19 and −9‰^25–27^. These values are widely used to study the source of soil organic matter. If the climate and environmental conditions are the same for all sampled plots in a given study, the variation in the soil isotope composition can be employed to measure the newly generated SOC after *S. alterniflora* invasion.

In this study, we hypothesized that time since *S. alterniflora* invasion would significantly affect the soil carbon pool in different soil fractions in the Chongming Dongtan tidal marsh. Analyzing the stable carbon isotopic composition and carbon concentration in the soil to a depth of 50 cm in areas where the invasive plant *S. alterniflora* and the native plant *P. australis* occurred allowed us to better understand the influence of invasive C_4_ plants on soil carbon. The major purposes of this study were to 1) understand the different vertical and horizontal distributions of the soil carbon pool in this Yangtze River estuary salt marsh; 2) quantify the contribution of *S. alterniflora* invasion to SOC; and 3) determine the influence of different *S. alterniflora* invasion times (3, 7 and 10 years) on the soil carbon pool.

## Results

### Plant biological traits

Along the southern transect, the aboveground biomass of the *S. alterniflora* community was 3500, 3889 and 3412 g m^−2^ for the low, middle and high tidal marshes, respectively, but these values did not differ significantly. These biomass values were approximately 1.5 times higher than those recorded for the *P. australis* community. Along the northern transect, the corresponding aboveground biomass of the *S. alterniflora* community was 3525, 2800 and 3104 g m^−2^ for the low, middle, and high tidal marshes, respectively; and these values were more than three times higher than those recorded for the *P. australis* community (Table 1).

**Table 1:** Biological parameters of *S. alterniflora* and *P. australis* communities in the Chongming Dongtan wetland, Shanghai, China. (n = 6).

Sampling site*	Community	Invasion time (years)	Aboveground biomass (g m^−2^)	Height (cm)	Stem density (stem m^−2^)
S-L-S	*S. alterniflora*	3	3500 ± 289	95 ± 3	359 ± 49
S-M-S	*S. alterniflora*	7	3889 ± 193	109 ± 3	500 ± 41
S-M-P	*P. australis*	—	1971 ± 236	225 ± 6	105 ± 17
S-H-S	*S. alterniflora*	10	3472 ± 82	110 ± 2	312 ± 58
S-H-P	*P. australis*	—	2468 ± 325	110 ± 2	69 ± 5
S-D-P	*P. australis*	—	2114 ± 176	234 ± 5	71 ± 2
N-L-S	*S. alterniflora*	3	3525 ± 449	118 ± 3	413 ± 58
N-M-S	*S. alterniflora*	7	2800 ± 85	134 ± 3	211 ± 37
N-M-P	*P. australis*	—	914 ± 50	181 ± 5	105 ± 11
N-H-S	*S. alterniflora*	10	3104 ± 106	117 ± 2	304 ± 8
N-H-P	*P. australis*	—	942 ± 75	166 ± 3	99 ± 5

*The first letter represents the transect, where S represents south, and N represents north. The second letter represents location, where L represents low marsh; M represents middle marsh; H represents high marsh; and D represents near dike. The third letter represents the plant, where S represents *S. alterniflora*, and P represents *P. australis*.

### Distribution patterns of SOC in Dongtan wetland

#### Horizontal distribution of SOC

**Figure 1:**
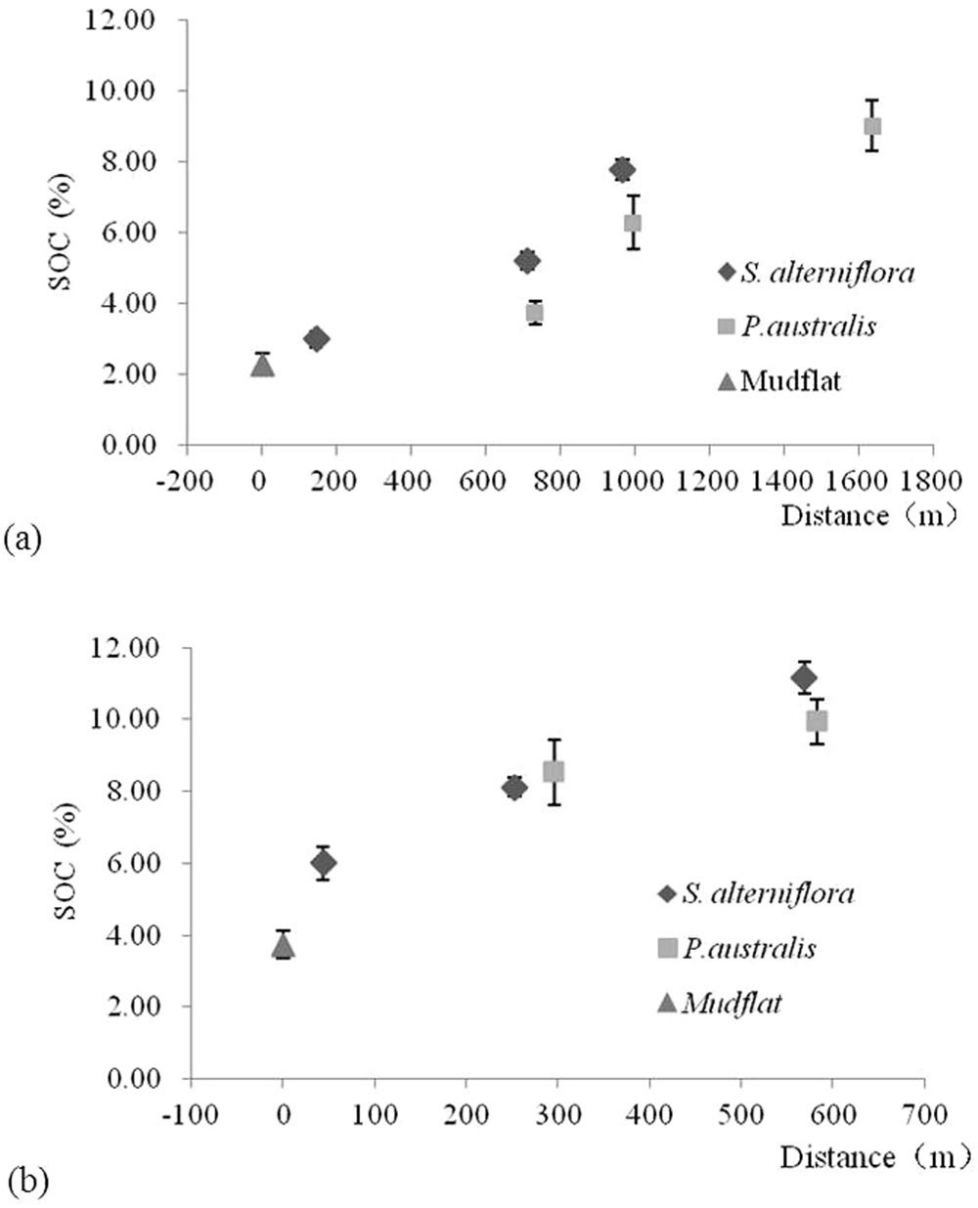
Spatial variation in the SOC content from the mudflats to the dike in the Chongming Dongtan wetland: (**a**) southern and (**b**) northern transects (n = 6).

Along the southern transect, the average SOC concentrations in all soil layers rapidly increased from approximately 2% to 8% across an area about 200 m from the mudflat to the vegetated tidal marsh which the distance reached 1000 m. Then, the SOC values remained at a high level, starting at approximately 8%, increased slightly when the distance reached approximately 1700 m. Along the entire northern transect, the soil total carbon content quickly increased rapidly from the mudflat to the vegetated area (Fig. 1).

Along the southern transect, the SOC of the *S. alterniflora* community increased during the period of plant invasion, ranging from 3.00% to 7.79%. Compared with the SOC of the mudflat, the SOC of the *S. alterniflora*-invaded plots (S-L-S) was increased by 128%. Additionally, the SOC of the *S. alterniflora*-invaded plots increased by 43.25% and 24.00% in the middle and high marshes, respectively, compared with that associated with *P. australis* at the same elevation. The SOC along the northern transect ranged from 3.87% to 11.16%. Compared with the SOC of the mudflat, the SOC of the *S. alterniflora*-invaded sample plots (N-L-S) was increased by 55.64%. The SOC measured in the *S. alterniflora* plots was generally higher than that in the *P. australis* plots, except in the N-M-S sample plot along the northern transect (Fig. 1b).

#### Vertical distribution of SOC

**Figure 2:**
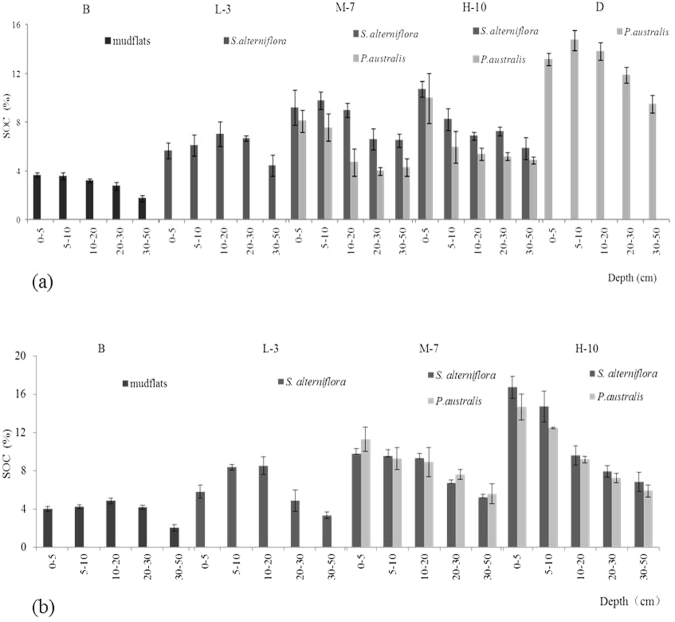
Spatial structure of SOC along the soil profile in the Chongming Dongtan wetland: (**a**) southern and (**b**) northern transects (n = 6).

The average SOC content in Dongtan wetland over a depth of 0–50 cm increased with increasing distance from the mudflat, but the rate of change varied in each soil layer. At the edge of the coastal marsh along the southern transect, the SOC content remained almost unchanged at a depths of 0–20 cm (*P* > 0.05) but decreased from 20 to 50 cm (*P* < 0.05) in the mudflat and the low and middle marshes (Fig. 2a). In the high marsh and near the dike, the SOC content decreased with increasing soil depth. The northern transect showed the same pattern (Fig. 2b). In addition, there was a greater decrease in SOC with depth in the high marsh than that in the low and middle marshes.

#### Isotope composition and soil carbon stabilization in Dongtan wetland

In this study, the δ^13^C value of *S. alterniflora* ranged from −13.68 to −14.5‰ (Table 2). The δ^13^C value of *S. alterniflora* leaves was much lower than those of the roots and litter (*P* < 0.05), while the δ^13^C values of the roots and litter were similar. The average δ^13^C value for the leaves, roots, and litter of −13.47‰, is regarded as representative for *S. alterniflora*.

**Table 2:** δ13C values of different *S. alterniflora* plant parts (n = 6).

Plant sample	Leaves	Roots	Litter
δ^13^C/‰	−13.72 ± 0.17	−13.48 ± 0.28	−13.20 ± 0.09

**Figure 3:**
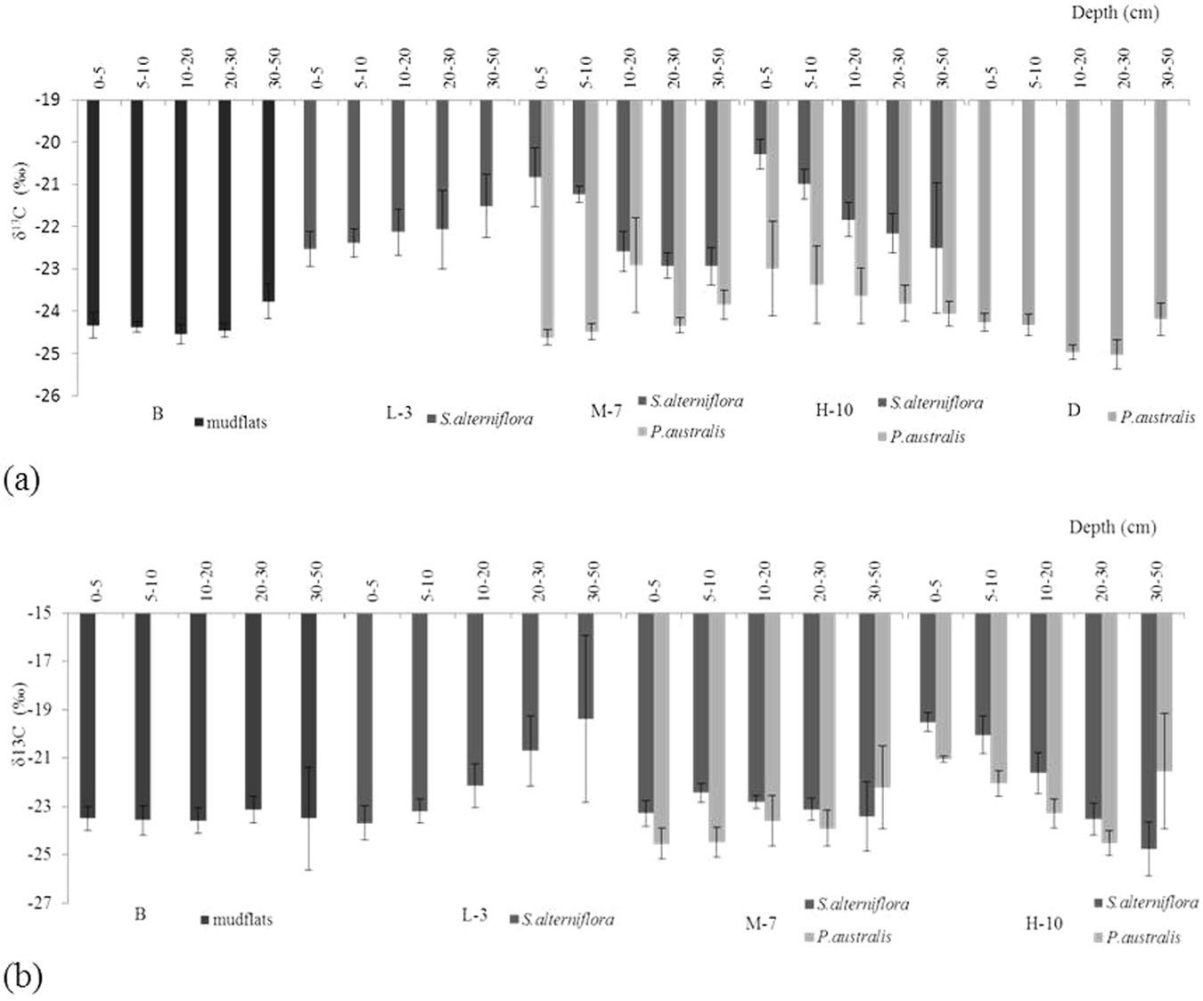
Variation in the δ^13^C values of SOC in *S. alterniflora* and *P. australis* communities: (**a**) southern and (**b**) northern transects (n = 6).

Along the southern transect, the δ^13^C value of the *S. alterniflora* community was less at greater soil depth in the low tidal marsh. The rate of increase of the δ^13^C value was 0.24 (*R*^2^ = 0.91, *P* < 0.05). In the middle and high tidal marshes, the δ^13^C value of the *S. alterniflora* community was more negative at greater soil depth. The rates of increase in the δ^13^C value were −0.59 (*R*^2^ = 0.88, *P* < 0.05) and −0.56 (*R*^2^ = 0.96, *P* < 0.05) for the middle and high tidal marshes, respectively. There were significant differences in the δ^13^C values between the *S. alterniflora* and *P. australis* communities (*P* < 0.05). Along the northern transect, the δ^13^C value of the *S. alterniflora* community of the mudflat and high tidal marsh exhibited the same pattern as for the southern transect, while the δ^13^C value of the *S. alterniflora* community did not change in the middle tidal marsh. In the high tidal marsh, the δ^13^C value of SOC in the *S. alterniflora* community was more negative as the stem density of *S. alterniflora* increased. The rate of decrease in the δ^13^C value was −1.16 (*P* < 0.05, *R*2 = 0.97). However, there were no significant differences in the δ^13^C values between the *P. australis* and *S. alterniflora* communities (Fig. 3).

#### Contribution of *S. alterniflora* to the soil carbon pool in Dongtan wetland

**Figure 4:**
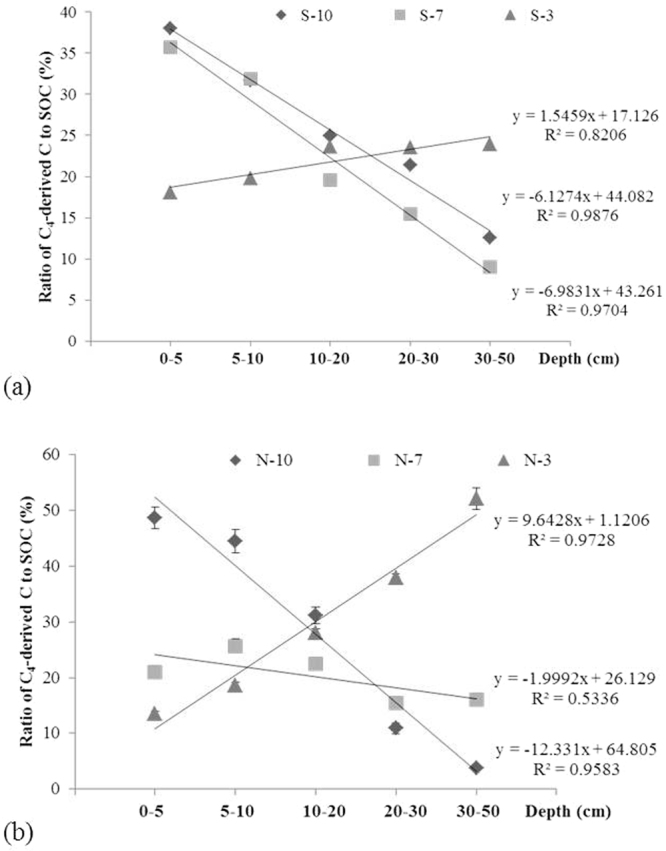
Contribution of carbon (C) derived from *S. alterniflora* to SOC: (**a**) southern and (**b**) northern transects (n = 6).

Along the southern transect, after 3 years of *S. alterniflora* invasion, the contribution of this specie to SOC was significantly positively correlated with soil depth (*P* < 0.01, *R*^2^ = 0.82); at soil depths of 0–5, 5–10, 10–20, 20–30 and 30–50 cm, the contribution of *S. alterniflora* to SOC represented 18.0, 19.8, 23.6, 23.6 and 23.8%, respectively. After 7 years of invasion, the SOC contribution of this specie was negatively correlated with soil depth (*P* < 0.01, *R*^2^ = 0.97); at soil depths of 0–5, 5–10, 10–20, 20–30 and 30–50 cm, the contribution to the SOC content were 35.7, 31.9, 19.6, 5.5 and 9.0%, respectively. After 10 years of invasion, the SOC contribution was significantly negatively correlated with soil depth (*P* < 0.01, *R*^2^ = 0.99); at soil depths of 0–5, 5–10, 10–20, 20–30 and 30–50 cm, the contributions of *S. alterniflora* to the SOC content were 38.0, 31.6, 25.0, 21.4 and 12.5%, respectively (Fig. 4a).

Along the northern transect, after 3 years of *S. alterniflora* invasion, the contribution of this species to the SOC content was significantly positively correlated with soil depth (*P* < 0.01, *R*^2^ = 0.97); at depths of 0–5, 5–10, 10–20, 20–30 and 30–50 cm, the contributions of this specie to the SOC content were 13.5, 18.6, 28.1, 37.9 and 52.2%, respectively. After 7 years of invasion, the contribution of *S. alterniflora* to the SOC content was not correlated with soil depth (*P* > 0.05, *R*^2^ = 0.53); at depths of 0–5, 5–10, 10–20, 20–30 and 30–50 cm, the contributions of this specie to the SOC content were 20.98, 25.68, 22.43, 15.46 and 16.09%, respectively. After 10 years of invasion, the contribution of this specie to the SOC content was significantly negatively correlated with soil depth (*P* < 0.01, *R*^2^ = 0.96); at depths of 0–5, 5–10, 10–20, 20–30 and 30–50 cm, the contributions of this specie to SOC was 48.7, 44.5, 31.1, 10.9 and 3.8%, respectively (Fig. 4b).

## Discussion

Plant invasion has been found to alter various components of nutrient cycles in wetland ecosystems^28^. Our results showed that the seaward invasion of *S. alterniflora* could play a major role in increasing carbon fixation in Chongming Dongtan wetland (Fig. 1). SOC was generally higher in soil dominated by *S. alterniflora* than that in soil dominated by native *P. australis* (Fig. 1). Higher SOC concentrations have been found in *S. alterniflora* communities due to high levels of rhizome and root biomass and elevated plant residue decomposition rates^15^. Our results showed that the aboveground biomass of *S. alterniflora* was significantly higher than that of *P. australis* (*P* < 0.05) (Table 1), indicating that *S. alterniflora* fixed more carbon and potentially provided more residues to the wetland soil^6,29,30^. In terms of the soil SOC distribution in the *S. alterniflora* community along the southern transect, the SOC content in the S-M-S plot was the highest but was not significantly different from that in the S-H-S plot (Fig. 2a). This showed that SOC stabilized after 7–10 years of *S. alterniflora* invasion. Wang *et al*.^31^ reported that SOC was saturated after 5–12 years of *S. alterniflora* invasion in marshes along the coast of Xinyanggang in North Jiangsu Province, China. However, SOC gradually increased with increasing time since invasion along the northern transect (Fig. 2b), similar to the results of previous research. Zhang *et al*.^11^ revealed that in Wanggang, Jiangsu Province, the carbon sequestration capacity of the topsoil remained unsaturated after 14 years of *S. alterniflora* invasion. The differences between the northern and southern transects could be related to the sedimentation conditions and organic carbon input from decomposition (e.g., the tidal influx rate and organic matter content in tidewater), environmental factors (e.g., soil moisture, redox potential, heat, bioturbation, and many associated factors) and the characteristics of species development in different research areas. Long-term monitoring of changes in SOC and controlled experiments are needed in future studies.

In the middle and high marshes, the SOC content was higher in the surface (0~20 cm) than that in the deeper soil layers. However, the highest SOC in the vegetation edge zone, the transition from the mudflat to the tidal marsh, occurred in the subsurface soil layer (5–20 cm) (Fig. 2). These results indicated that a considerable amount of partly decomposed *S. alterniflora* residue remained within soil depths of 5–20 cm. At the vegetation edge zone, the soil formation process began on the dead *S. alterniflora* trapped by the tidally transported sediment. Farther from the mudflat, tidal forcing is weak, resulting in less sediment deposited on the surface and a higher SOC content in the near-surface layer. Aboveground parts (i.e., standing desiccated parts) decompose in the air or fall to the ground. The tide carries away decomposed soluble carbon and tiny carbon granules, transporting them inshore, where they are deposited on the soil surface or leach into the soil^6,32,33^.

The photosynthetic pathways of different plants make different relative contributions to the net primary productivity of surface soil, and the δ^13^C values of SOC vary^8^. Therefore, under conditions of long-term stability of plant species, the δ^13^C value of SOC is strongly influenced by plant δ^13^C^26,33–35^. Our stable isotopic analysis suggested that the δ^13^C value of SOC along the soil profile differed between the *S. alterniflora* and *P. australis* communities. The δ^13^C value was less negative at greater depth, which was related to the contribution from ^13^C-enriched carbon sources, after 3 years of invasion by *S. alterniflora* (Fig. 3). This finding is consistent with previously reported δ^13^C distributions in soil profiles^36,37^. However, after 7 and 10 years, the δ^13^C value was typically more negative at greater depth in both *S. alterniflora* and *P. australis* communities. This result can be explained by the presence of SOC derived from ^13^C-depleted C_3_ vegetation^32^. Cheng *et al*.^15^ considered that ^13^C-depleted C_4_ *S. alterniflora* contributed less to SOC than native species in Jiuduansha tidal marsh because of a short invasion (i.e.,<7 years) before which *Scirpus maiqueter* monoculture was dominant on the island for a 30-year period.

Along the southern transect, the rates of the decrease in δ^13^C values along the 0–20 cm soil profile in the *S. alterniflora* community were 0.88 (*P* < 0.01, *R*^2^ = 0.99) and 0.78 (*P* < 0.05, *R*^2^ = 0.80) for the middle and high marshes, respectively. In addition, the rates of the decrease along the 20–50 cm soil profile in the *S. alterniflora* community were 0.18 (*P* < 0.05, *R*^2^ = 0.80) and 0.33 (*P* < 0.01, *R*^2^ = 0.99), respectively. These results likely occurred because SOC decomposes rapidly in surface soil. The SOC was almost completely decomposed when the δ^13^C value reached a plateau. Subsequently, the decomposition rate of SOC slowed, as did the change in the δ^13^C value. In this study, the *S. alterniflora* residue contributed predominantly to SOC at a depth of 20 cm compared with a lower contribution to surface SOC due to increased oxygenation that resulted in greater decomposition of organic carbon^38^. *S. alterniflora* contributed less to deep SOC because of less root exudation and weaker osmosis of soluble organic carbon^34^.

**Figure 5:**
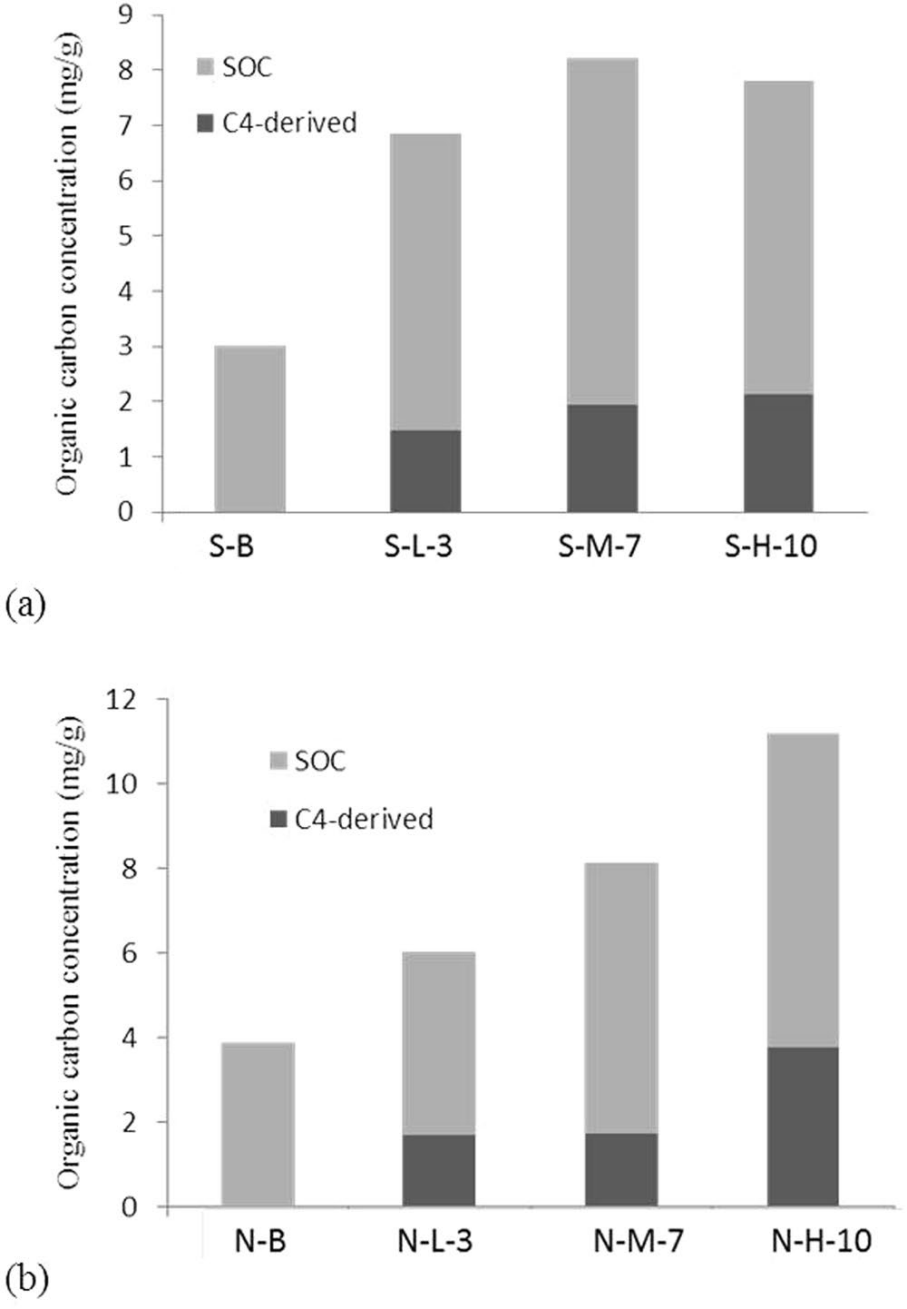
Relationships between the proportions and contents of *S. alterniflora* sources and invasion time: (**a**) southern and (**b**) northern transects.

In the present study, the sediment of the mudflat originated mainly from tidally transported sediment, which is likely why the δ^13^C value of the mudflat sediments was approximately −24.33‰, which is similar to that of terrestrial C_3_ plants, such as *P. australis*. A double-variable hybrid model was used to calculate the contribution of *S. alterniflora* to surface SOC; with increasing time since *S. alterniflora* invasion, the content and proportion of the contributed SOC increased (Fig. 5). During the seaward invasion of *S. alterniflora*, the SOC content and proportion attributed to this species showed a clear positive correlation with time since invasion (*P* < 0.01), revealing that the SOC contributed by *S. alterniflora* increased continuously. The proportion of SOC contributed by *S. alterniflora* over 10 years of invasion reached 27.3 and 33.7% along the southern and northern transects, respectively.

A study conducted in the Jiuduansha tidal marsh in Shanghai showed that after 8 years of seaward invasion by *S. alterniflora* in a *S. mariqueter* community, the contribution to surface SOC ranged from 5 to 10%^15^. In addition, after 14 years of landward invasion by *S. alterniflora* in a *Suaeda salsa* community in the Wanggang tidal marsh in Jiangsu Province, the contribution of *S. alterniflora* to surface SOC was 18.7%^31^. First of all, soil organic carbon input was affected by the high primary productivity and extensive root system of *S. alterniflora*. In addition, *S. alterniflora* had higher primary productivity in Chongming Dongtan, ranging from 2800 to 3889 g·m^−2^, compared with that in Wanggang and Jiuduansha tidal marshes, which ranged from 1442 to 2596 g·m^−2 ^^39,40^. It can immobilize carbon from the atmosphere and surrounding areas during growth; this carbon then enters the soil via litter and root secretions, which can directly influence the SOC content. And its well-developed roots, *S. alterniflora* can weaken the power of the tide, solidifying sediments in the flat and sedimenting tiny granules, which greatly improves adsorption due to the high specific surface area of the tiny granules, facilitating an increase in SOC. Second, our research site is located in the Yangtze River estuary, which carried a large number of organic matters through the tidal action, and deposited in Chongming Dongtan tidal marsh. Wang *et al*.^31^ considered the biomass of *S. alterniflora* and the sediment particle size to be the dominant factors determining the content of SOC. Additionally, the SOC content derived from *S. alterniflora* showed little correlation with *S. alterniflora* biomass but presented a clear positive correlation with particle size, which revealed a complicated process between the plant and soil that was greatly affected by soil characteristics.

The organic carbon input of salt marsh ecosystem mainly has two kinds of ways, including endogenous and exogenous input. Endogenous input contains soil organic matter and litter decomposition^41^. The exogenous inputs include suspended particles and dissolved organic carbon brought by tidal waters^42^. In Chongming Dongtan, suspended organic matter and sediment entered the soil carbon pool through horizontal flux. *S. alterniflora* has dense stalks and developed underground roots. Its ability to attenuate wave energy and reduce flow contributes to the sedimentation and deposition of silt^43^. Therefore, in this study of the effects of invasion on the carbon balance of ecosystem, the lateral flux caused by tidal activity is a component as well.

In this study, the tide had more influence on the contribution on soil carbon pool during the early period of invasion (after 3 years of invasion). With invasion time extension (after 7 years of invasion), the increase of soil carbon pool mainly depends on the plant (Fig. 4). The tidal flow is affected by surface roughness, elevation, and friction from plant stems and leaves^44^. After 3 years of invasion, the *S. alterniflora* patch adjacent to the bare flat had less tidal energy consumption and greater biomass loss (such as litter and lodged plants) compared with observations recorded after 7 and 10 years of invasion. In coastal marsh, the frequent cyclical effects of tide brought a non-negligible sea source input to soil carbon pool and showed an influence on the early stage of *S. alterniflora* invasion^45^.

## Conclusions

In summary, we found that *S. alterniflora* invasion in Chongming Dongtan wetland over the past 10 years was associated with increased SOC concentrations. SOC was generally higher in the *S. alterniflora* than in the native *P. australis* community. The contribution of *S. alterniflora* to the soil carbon pool exhibited an obvious gradient in terms of its horizontal distribution. In the high marsh, which was invaded 10 years ago, the contribution was positively correlated with soil depth, while in the low marsh, which was invaded by *S. alterniflora* within the past 3 years, the contribution of *S. alterniflora* to the soil carbon pool was negatively correlated with depth. This result might have occurred because litter is frequently washed away by the tide in the low marsh. Thus, the main source of SOC is likely the root exudates of *S. alterniflora*.

## Materials and Methods

### Study area

**Figure 6:**
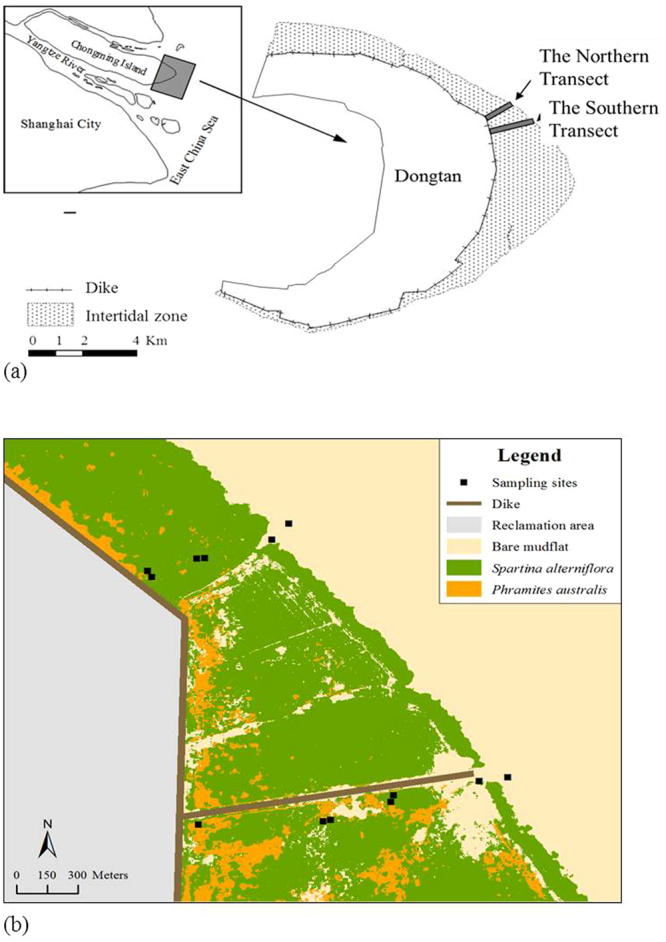
(**a**) Location of the sampling site in Chongming Dongtan, Shanghai, China. (**b**) Map of the soil profile sampling sites. Erdas9.2 TM was used to acquire the spatial distribution of plant communities from the purchased remote sensing image, which came from the 2012.5.16 ZY-02C satellite (HRC 2.36 meters), and ArcGIS10.2 TM was subsequently employed to draw these images.

This study was conducted in the Shanghai Chongming Dongtan Wetland National Nature Reserve (31°25′–31°38′N, 121°50′–122°05′E) located at the eastern end of Chongming Island. Chongming Dongtan is formed by alluvial silt and soil from the upper reaches of the Yangtze River and is the largest and youngest estuarine tidal marsh in the Yangtze River estuary. Chongming Dongtan has a northern subtropical ocean climate, with average annual precipitation of 1117.1 mm and a mean annual temperature of 15.3 °C (Fig. 6a). The native species *Scirpus mariqueter* and *Phragmites australis* have dominanted for over 20 years, while invasive *S. alterniflora* has rapidly spread in this tidal marsh and is replacing native species due to its considerable seed production and high germination rate^46^. Currently, *P. australis* and *S. alterniflora* now show zonation in their distribution in Chongming Dongtan^46^.

Using ZY-3 satellite high-resolution remote sensing images and GPS coordinates, two transects were established using a combination of factors specific to each site, such as vegetation type, invasion time, water content, soil physical factors (pH, ORP, Salinity) (Table 3). The southern and northern transects were located in southeastern and northeastern Dongtan, respectively. The salt marsh directly landward of the study area was reclaimed in 1998. Both transects were established so that they extended through a variety of plant communities containing *S. alterniflora* and *P. australis*. The southern transect–with westernmost geographic coordinates of 31°50′N and 121°13′E and easternmost coordinates of 31°32′N and 121°58′E – was approximately 1800 m long. The northern transect–with westernmost coordinates of 31°91.67ʹN and 121°08.85′E and easternmost coordinates of 31°90.77′N and 121°95.52′E–was approximately 700 m long. Both transects were divided into three parts according to the tidal characteristics, i.e., low, middle and high tidal marshes. Low marsh refers to areas of the saltmarsh below mean high water level of neap tide. Middle marsh refers to areas of the saltmarsh between mean water level and mean high water level. High marsh refers to areas of the saltmarsh above mean high water. According to the elevations of two tidal gauges near Chongmin Dongtan (Jigujiao Gauge and Wusong Gauge), the mean high water level of Chongming Dongtan is 1.46 ± 0.01 m, the mean high water level during neap tide is 0.95 ± 0.05 m, and the mean water level is approximately 0.2 ± 0.03 m. These two chronosequenced transects extended through remnant *P. australis* and the *S. alterniflora* communities that replaced *P. australis* in 2001 (10 years previously), 2004 (7 years) and 2008 (3 years). Plots were established every 200–300 m along the transects. Each plot consisted of two 20 m × 20 m quadrats, and samples were collected from five replicate 0.5 m × 0.5 m subquadrats randomly established within each quadrat (Fig. 6b).

**Table 3:** Parameters of sampling sites of *S. alterniflora* and *P*.

Sampling site*	Community	Invasion time (years)	Water (%)	ORP	pH	Salinity (‰)
S-L-S	*S. alterniflora*	3	37.5	−176.3	7.20	14.3
S-M-S	*S. alterniflora*	*7*	32.1	−53.2	7.44	18.3
S-M-P	*P. australis*	—	30.3	−44.7	7.37	17.1
S-H-S	*S. alterniflora*	10	31.5	−13.0	7.53	25.4
S-H-P	*P. australis*	—	31.8	8.2	7.54	18.3
S-D-P	*P. australis*	—	35.2	−78.1	7.32	10.1
N-L-S	*S. alterniflora*	3	38.8	−203.3	7.23	16.9
N-M-S	*S. alterniflora*	7	32.6	−163.6	7.40	20.3
N-M-P	*P. australis*	—	31.5	−151.1	7.35	17.6
N-H-S	*S. alterniflora*	10	30.1	−82.1	7.57	30.1
N-H-P	*P. australis*	—	29.4	−51.3	7.45	19.6

*australis* communities in the Chongming Dongtan wetland, Shanghai, China (n = 6).

### Field sample collection

Soil, leaf, litter, and root samples were collected from the *S. alterniflora* and *P. australis* communities. Cores were extracted using a stainless-steel peat core sampler and were divided into 5 cm sections in the field. Each section was sealed in multiple plastic bags with as much air squeezed out as possible and stored on ice or at 4 °C to avoid volatilization losses and minimize microbial activity until analysis.

### Laboratory analyses

Plant biomass was sampled in all plots in September 2011, when the aboveground plant biomass was the greatest according to a previous study in a nearby wetland^47^. All aboveground plant biomass was harvested and then oven-dried at 50 °C to a constant weight.

Plant material and organic debris were first gently removed from the soil samples using forceps, and the leaf, root, litter, and soil samples were then dried at 60 °C to a constant weight and ground to pass through 100-mesh sieves^34,48^. After thorough mixing, approximately 10 g of dried soil was treated with 0.5 mol L^−1^ hydrochloric acid (HCl) for 24 h at room temperature to remove any inorganic carbon, and the unhydrolyzed residue was categorized as SOC^48^. Approximately 3 mg of the leaf, root, and litter subsamples was weighed and analyzed to determine the total carbon contents of the plant materials. Non-HCl-treated soil subsamples (approximately 50 mg) were weighed and analyzed to determine soil total carbon. These measurements were performed using a soil element analyzer (Flash, EA, 1112 Series, USA).

The carbon isotope ratio of organic materials (leaves, roots, litter and HCl-treated soil) was measured using an isotope ratio mass spectrometer (Thermo Fisher, Delta V Advantage, Flash, EA, 1112 Series, USA). The precision of δ^13^C was ±0.1‰, which was based on repeated measurements of a laboratory working standard. The δ^13^C value of the organic matter was calculated using the following equation:

1 $${{\rm{\delta }}}^{13}{\rm{C}}=[{{\rm{R}}}_{{\rm{sample}}}/{{\rm{R}}}_{{\rm{PDB}}}-1]\times 1000$$

where R = ^13^C/^12^C, and PDB indicates Pee Dee Belemnite – the belemnite carbonate standard of the Peedee Formation in South Carolina, USA^49,50^.

### Calculation of carbon inputs

The proportion f is the proportion of *S. alterniflora*-derived carbon in the soil. This calculation was based on the δ^13^C values of the soil before and after *S. alterniflora* invasion (δ^13^C_old_ and δ^13^C_new_, respectively) and the δ^13^C value of *S. alterniflora* (δ^13^C_mix_), which was the average of the values for the leaves, roots, and litter (Table 2)^15,34^.

2 $${{\rm{\delta }}}^{13}{{\rm{C}}}_{{\rm{new}}}={\rm{f}}\times {{\rm{\delta }}}^{13}{{\rm{C}}}_{{\rm{mix}}}+(1-{\rm{f}})\times {{\rm{\delta }}}^{13}{{\rm{C}}}_{{\rm{old}}}$$

3 $${\rm{f}}=({{\rm{\delta }}}^{13}{{\rm{C}}}_{{\rm{new}}}-{{\rm{\delta }}}^{13}{{\rm{C}}}_{{\rm{old}}})/({{\rm{\delta }}}^{13}{{\rm{C}}}_{{\rm{mix}}}-{{\rm{\delta }}}^{13}{{\rm{C}}}_{{\rm{old}}})\times 100 \% $$

### Statistical analyses

Statistical analyses were performed using the statistical software package SPSS version 18 for Windows 7. Data were tested for normality prior to statistical analysis. One-way ANOVA at a 5% significance level was employed to determine the differences in SOC and the biomass, height and density of the plants. Significant differences in plant δ^13^C values between the two species were analyzed using the t-test and the least significant difference at *P* < 0.05. δ^13^C data were arcsine transformed to ensure homoscedasticity before the ANOVAs were conducted. Regression analyses were performed to test the relationships between the accumulative rates of SOC or *S. alterniflora*-derived carbon and the ages of the plant invasions.

### Data availability statement

The datasets generated during the current study are available from the corresponding author upon reasonable request.

## References

[1] Deegan LA (2012). Coastal eutrophication as a driver of salt marsh loss. Nature.

[2] McLeod E (2011). A blueprint for blue carbon: toward an improved understanding of the role of vegetated coastal habitats in sequestering CO_2_. Front. Ecol. Environ..

[3] Mitsch WJ, Gosselink JG (2000). The value of wetlands: importance of scale and landscape setting. Ecol. Econ..

[4] Duarte CM, Middelburg JJ, Caraco N (2005). Major role of marine vegetation on the oceanic carbon cycle. Biogeosciences.

[5] Hopkinson CS, Cai WJ, Hu XP (2012). Carbon sequestration in wetland dominated coastal systems - a global sink of rapidly diminishing magnitude. Curr. Opin. Environ. Sustain..

[6] Liao C (2008). Altered ecosystem carbon and nitrogen cycles by plant invasion: a meta-analysis. New Phytologist.

[7] Chmura GL, Anisfeld SC, Cahoon DR, Lynch JC (2003). Global carbon sequestration in tidal, saline wetland soils. Glob. Biogeochem. Cycle.

[8] Gebrehiwet T, Koretsky CM, Krishnamurthy RV (2008). Influence of *Spartina* and *Juncus* on saltmarsh sediments. III. Organic geochemistry. Chem. Geol..

[9] Allison SD, Vitousek PM (2004). Rapid nutrient cycling in leaf litter from invasive plants in Hawai’i. Oecologia.

[10] Ehrenfeld JG (2003). Effects of exotic plant invasions on soil nutrient cycling processes. Ecosystems.

[11] Zhang Y, Ding W, Luo J, Donnison A (2010). Changes in soil organic carbon dynamics in an Eastern Chinese coastal wetland following invasion by a C_4_ plant *Spartina* alterniflora. Soil Biology and Biochemistry.

[12] Qin, P. & Zhong, C. X. *Applied Studies on Spartin*a. (Ocean Press, 1992).

[13] Bruno JF (2000). Facilitation of cobble beach plant communities through habitat modification by *Spartina* alterniflora. Ecology.

[14] Peng, S. & Xiang, Y. The invasion of exotic plants and effects of ecosystems. *Acta Ecologica Sinica* **19**, 560–569 (In Chinese with English Abstract) (1999).

[15] Cheng X (2006). Short-term C_4_ plant *Spartina alterniflora* invasions change the soil carbon in C_3_ plant-dominated tidal wetlands on a growing estuarine Island. Soil Biology and Biochemistry.

[16] Benner R, Fogel ML, Sprague EK (1991). Diagenesis of belowground biomass of *Spartina alterniflora* in salt‐marsh sediments. Limnol. Oceanogr..

[17] Currin CA, Newell SY, Paerl HW (1995). The role of standing dead *Spartina* alterniflora and benthic microalgae in salt marsh food webs: Considerations based on multiple stable isotope analysis. Mar. Ecol.-Prog. Ser..

[18] Dai JH, Sun MY (2007). Organic matter sources and their use by bacteria in the sediments of the Altamaha estuary during high and low discharge periods. Org. Geochem..

[19] Ember LM, Williams DF, Morris JT (1987). Processes that influence carbon isotope variation in salt marsh sediments. Mar. Ecol.-Prog. Ser..

[20] Fogel ML, Sprague EK, Gize AP, Frey RW (1989). Diagenesis of organic matter in Georgia salt marshes. Estuar. Coast. Shelf Sci..

[21] Gardner LR (1990). Simulation of the diagenesis of carbon, sulfur, and dissolved oxygen in salt marsh sediments. Ecol. Monogr..

[22] Middelburg JJ, Nieuwenhuize J, Lubberts RK, van de Plassche O (1997). Organic carbon isotope systematics of coastal marshes. Estuar. Coast. Shelf Sci..

[23] Zhang SP (2011). Organic carbon accumulation capability of two typical tidal wetland soils in Chongming Dongtan, China. J. Environ. Sci..

[24] Boschker HTS, de Brouwer JFC, Cappenberg TE (1999). The contribution of macrophyte-derived organic matter to microbial biomass in salt-marsh sediments: Stable carbon isotope analysis of microbial biomarkers. Limnol. Oceanogr..

[25] Bernoux M, Cerri CC, Neill C, de Moraes JFL (1998). The use of stable carbon isotopes for estimating soil organic matter turnover rates. Geoderma.

[26] Nyberg G, Ekblad A, Buresh RJ, Högberg P (2000). Respiration from C_3_ plant green manure added to a C_4_ plant carbon dominated soil. Plant Soil.

[27] Staddon PL (2004). Carbon isotopes in functional soil ecology. Trends Ecol. Evol..

[28] Ehrenfeld, J. G. *Plant-soil interactions. In*: Levin, S. *(Ed.), Encyclopedia of Biodiversity*. 689–707 (Academic Press, 2001).

[29] Long, S. P. *Evnironmental responses. In: Sage*, R.F.*, Monson*, R.K. *(Eds.)*, *C*_4_*Plant Biology*. 215–249 (Academic Press, 1999).

[30] Still CJ, Berry JA, Ribas-Carbo M, Helliker BR (2003). The contribution of C_3_ and C_4_ plants to the carbon cycle of a tallgrass prairie: an isotopic approach. Oecologia.

[31] Wang, G., Yang, W., Wang, G., Liu, J. e. & Hang, Z. The effects of *Spartina alterniflora* seaward invasion on soil organic carbon fractions,sources and distribution. *Acta Ecologica Sinica* **33**, 2474–2483 (In Chinese with English Abstract) (2013).

[32] Henderson DC, Ellert BH, Naeth MA (2004). Utility of C-13 for ecosystem carbon turnover estimation in grazed mixed grass prairie. Geoderma.

[33] Hobbie EA, Johnson MG, Rygiewicz PT, Tingey DT, Olszyk DM (2004). Isotopic estimates of new carbon inputs into litter and soils in a four-year climate change experiment with Douglas-fir. Plant Soil.

[34] Chiang PN, Wang MK, Chiu CY, King HB, Hwong JL (2004). Changes in the grassland-forest boundary at Ta-Ta-Chia long term ecological research (LTER) site detected by stable isotope ratios of soil organic matter. Chemosphere.

[35] Osher LJ, Matson PA, Amundson R (2003). Effect of land use change on soil carbon in Hawaii. Biogeochemistry.

[36] Ehleringer JR, Buchmann N, Flanagan LB (2000). Carbon isotope ratios in belowground carbon cycle processes. Ecol. Appl..

[37] Follett RF (1997). Carbon isotope ratios of great plains soils and in wheat-fallow systems. Soil Sci. Soc. Am. J..

[38] Chen XP (2010). Evaluating the impacts of incubation procedures on estimated *Q*_10_ values of soil respiration. Soil Biol. Biochem..

[39] Bu NS. Soil CO2, CH4 emissions and carbon dynamic in wetland of Yangtza Estuary. Doctor Disseration, Fudan University, Sahnghai. 25 (2012).

[40] Feng ZX (2015). The response of organic carbon content to biomass dynamics in Spartina alterniflora marsh. Acta Ecologica Sinica.

[41] Hr H, Mannino A (2001). The chemical composition and cycling of particulate and macromolecular dissolved organic matter in temperate estuaries as revealed by molecular organic tracers. Organic Geochemistry.

[42] Hemminga MA, Cattrijsse A, Wielemaker A (1996). Bedload and nearbed detritus transport in a tidal saltmarsh creek. Estuarine, Coastal and Shelf Science.

[43] Liao CZ (2007). Invasion of Spartina alterniflora enhanced ecosystem carbon and nitrogen stocks in the Yangtze Estuary, China. Ecosystems.

[44] Shi BW, Yang SL, Luo XX, Xu XJ (2010). A wave attenuation over the transitional zone of mudflat and salt marsh: A case study in the eastern Chongming on the Changjiang delta. Acta Oceanologica Sinica.

[45] Wang D, Zhang R, Xiong J, Guo HQ, Zhao B (2015). Contribution of invasive species Spartina alterniflora to soil organic carbon pool in coastal wetland: Stable isotope approach. Chinese Journal of Plant Ecology.

[46] Li H, Zhang L, Wang D (2006). Distribution of an exotic plant *Spartina alterniflora* in Shanghai. Biodiversity Science.

[47] Cheng X (2008). Assessing the effects of short-term *Spartina alterniflora* invasion on labile and recalcitrant C and N pools by means of soil fractionation and stable C and N isotopes. Geoderma.

[48] Lin GH, Ehleringer JR, Rygiewicz PT, Johnson MG, Tingey DT (1999). Elevated CO_2_ and temperature impacts on different components of soil CO_2_ efflux in Douglas-fir terracosms. Glob. Change Biol..

[49] Desjardins T, Carneiro A, Mariotti A, Chauvel A, Girardin C (1996). Changes of the forest-savanna boundary in Brazilian Amazonia during the Holocene revealed by stable isotope ratios of soil organic carbon. Oecologia.

[50] Wooller M, Smallwood B, Jacobson M, Fogel M (2003). Carbon and nitrogen stable isotopic variation in Laguncularia racemosa (L.) (white mangrove) from Florida and Belize: implications for trophic level studies. Hydrobiologia.

